# 

**Published:** 1858-07

**Authors:** 


NORTH AMERICAN

### MEDICO-CHIRURGICAL REVIEW.

Vol. II.] JULY, 1858. [No. 4.

### CONTENTS.

### ANALYTICAL AND CRITICAL REVIEWS.

ART. PAGE

I.—Lectures on the Diseases of Women. By CHARLES WEST, M.D. . 593

II.—Leçons sur le Chancre. By Dr. RICORD . . . 606

III.—Clinical Lectures on the Principles and Practice of Medicine. By JOHN HUGHES BENNETT, M.D. . . . . . 618

IV.—Silver Sutures in Surgery. By J. MARION SIMS, M.D. . . 635

V.—Lectures on the Principles and Practice of Physic. By THOMAS WATSON, M.D. . . . . . . . . . . . 653

VI.—Contributions to Operative Surgery and Surgical Pathology. By J. M. CARNOCHAN, M.D. . . . . . . . . 656

### ORIGINAL COMMUNICATIONS.

I.—Practical Observations on the Nature and Treatment of Tuberculosis of the Hip-Joint. By S. D. GROSS, M.D. . . . . 658

II.—Removal of an Ovarian Tumor, in which the Écraseur was used. By JOHN L. ATLEE, M.D. . . . . . . . . . 698

III.—Experiments in Reference to Section II. of a Paper in the "May No. of the Review," on "The Perceptive Power of the Spinal Cord." By GEORGE R. PATON, M.D. . . . . . . 703

IV.—Proceedings of the Pathological Society of Philadelphia . . 705

### AMERICAN PROGRESS OF MEDICAL SCIENCE.

Report on Practical Surgery. PART II. By EDWARD HARTSHORNE, M.D., One of the Surgeons of Wills' Hospital, Philadelphia . . . . 732

### BI-MONTHLY FOREIGN ABSTRACTS.

NOVELTIES IN MEDICAL, SURGICAL, OBSTETRICAL, AND CHEMICAL SCIENCE; CHIEFLY FROM THE FRENCH, GERMAN, AND ITALIAN. BY EMIL FISCHER, M.D.

PHYSIOLOGY.

I.—Experimental Researches on the Physiological Properties and Uses of the Red and of the Black Blood. By E. BROWN-SÉQUARD, M.D. . 761

ART. PAGE

II.—Memoir on the Effects of Extirpation of the Pancreas. By Prof. Bérard and M. Colin . . . . . . . . . . 765

III.—On the Nervous Centres controlling the Contraction of the Uterus. By O. Spiegelberg, M.D. . . . . . . . . . 767

IV.—On the Peristaltic Motion of the Intestines. By O. Spiegelberg, M.D. . . . . . . . . . . . . 769

PATHOLOGY.

V.—On Spontaneous Primary Pyæmia. By Prof. C. A. Wunderlich . . . . . . . . . . . . 770

Editors' Table.—American Medical Association . . . . . . 775

Medical Society of the State of Pennsylvania . . . . . . 778

Consultations with Homœopaths . . . . . . . . 779

Resignations and Appointments in Medical Schools . . . . . 780

Shelby Medical College . . . . . . . . . . 781

Inauguration of the Jenner Monument . . . . . . . 781

Bi-Monthly Abstracts . . . . . . . . . . 781

Junior Editor of the Review . . . . . . . . . 782

Necrological Notices.—Dr. Robert Hare . . . . . . . 782

Dr. Johannes Muller . . . . . . . . . . . 783

Prof. L. W. Mauthner . . . . . . . . . . . 783

Dr. William Gregory . . . . . . . . . . . 783

Dr. Robert Harrison . . . . . . . . . . . 783

Bi-Monthly Bibliographical Record . . . . . . . . 784

